# Stability Improvement of Perovskite Solar Cells by the Moisture-Resistant PMMA:Spiro-OMeTAD Hole Transport Layer

**DOI:** 10.3390/polym14020343

**Published:** 2022-01-17

**Authors:** Shaohua Ma, Shangzheng Pang, Hang Dong, Xiaoping Xie, Gang Liu, Peng Dong, Dawei Liu, Weidong Zhu, He Xi, Dazheng Chen, Chunfu Zhang, Yue Hao

**Affiliations:** 1 Key Laboratory of Micro/Nano Systems for Aerospace, Ministry of Education, Northwestern Polytechnical University, Xi’an 710072, China; mashaohua@spic.com.cn; 2 Wide Bandgap Semiconductor Technology Disciplines State Key Laboratory, Shaanxi Joint Key Laboratory of Graphene, School of Microelectronics, Xidian University, Xi’an 710071, China; szpang@stu.xidian.edu.cn (S.P.); donghangxd@163.com (H.D.); wdzhu@xidian.edu.cn (W.Z.); hxi@xidian.edu.cn (H.X.); yhao@xidian.edu.cn (Y.H.); 3 Qinghai Huanghe Hydropower Development Co., Ltd., Xining 810008, China; xiexiaoping@spic.com.cn (X.X.); liugang@spic.com.cn (G.L.); dongpeng@spic.com.cn (P.D.); liudawei02@spic.com.cn (D.L.)

**Keywords:** perovskite solar cells, hole transport layer, spiro-OMeTAD, PMMA, stability

## Abstract

Perovskite solar cells (PSCs) based on the 2,2′,7,7′-tetrakis(N,N-di-p-methoxyphenylamine)-9,9′-spirobifluorene (spiro-OMeTAD) hole transport layer have exhibited leading device performance. However, the instability caused by this organic function layer is a very important limiting factor to the further development of PSCs. In this work, the spiro-OMeTAD is doped with polymethyl methacrylate (PMMA), which is further used as the hole transport layer to improve the device stability. It is shown that the PMMA can effectively improve the moisture and oxygen resistance of spiro-OMeTAD, which leads to improved device stability by separating the perovskite layer from moisture and oxygen. The device efficiency can maintain 77% of the original value for PSCs with the PMMA-doped spiro-OMeTAD hole transport layer, under a natural air environment (RH = 40%) for more than 80 days. The results show that the moisture- and oxygen-resistant PMMA:spiro-OMeTAD hole transport layer is effective at improving the device performance.

## 1. Introduction

With the improvement in power conversion efficiency (PCE) from 3.8% to over 25% in only a few years, perovskite solar cells (PSCs) are rapidly emerging as a new type of photovoltaic device in the photovoltaic society [1]. The dramatically increased performance is mainly attributed to the unique perovskite material properties, such as the unusual carrier diffusion length, strong light harvesting capability, and ambipolar charge transport properties [2].

In general, PSCs can be divided into the regular structure with the NIP framework, and the inverted structure with the PIN framework. Both structures have helped PSCs to achieve great progress, with PCEs exceeding 20% in the past few years. However, up to now, most of the record efficiencies have been obtained based on the regular structure, because this structure has better energy level matching, and more efficient charge transport and extraction [3,4]. In the regular structure, MAPbI_3_ is a traditional perovskite light absorption material. At the same time, TiO_2_ and 2,2′,7,7′-tetrakis(N,N-di-p-methoxyphenylamine)-9,9′-spirobifluorene (spiro-OMeTAD) are usually used as electron and hole transport materials (ETM and HTM), respectively. Based on the structure of TiO_2_/MAPbI_3_/spiro-OMeTAD, PSCs have acquired satisfactory device efficiency. However, PSCs still suffer from the device stability issue. To overcome device degradation, many efforts have been put into improving the stabilities of both the material and structure. The intrinsic instability of MAPbI_3_ is one important factor influencing the long-term storage of PSCs, and is caused by its soft nature and low lattice formation energy. To acquire better chemical stability, Cs^+^, FA^+^, Br^−^, and other ions are added into the perovskite framework to replace MA^+^ and I^−^ [5,6], which has efficiently improved the intrinsic stability of perovskite film. However, external stressors, such as heat, oxygen, light, and especially moisture, are also important factors that damage PSCs [7,8,9]. The electron and hole transport layers (ETL and HTL) not only transport carriers, but also protect the perovskite film by isolating it from external stressors.

Although there are many HTL materials, such as CuI [10], CuSCN [11], poly(3,4-ethylenedioxythiophene):poly(styrene sulfonate) (PEDOT:PSS) [12], poly 3-hexylthiophene) (P3HT) [13], polytriarylamine (PTAA) [14], and 2,2′,7,7′-tetrakis (N,N-di-p-methoxyphenylamine)-9,9′-spirobifluorene (spiro-OMeTAD), spiro-OMeTAD has been adopted the most. Spiro-OMeTAD is an ideal hole transport material (HTM), which possesses optimum conductivity with the addition of lithium bis (tri-fluoromethane) sulfonimide (LiTFSI), tris(2-(1H-pyrazol-1-yl)-4-tert-butylpyridine)-cobalt(III)-tris(bis(trifluoromethylsulfonyl)imide)) FK 209 Co(III), TFSI salt, 4-tert-butylpyridine (t-BP), and air exposure [10]. Many reports have shown that PSCs based on the spiro-OMeTAD HTL have realized high PCEs over 21% [15,16]. However, spiro-OMeTAD cannot effectively separate the perovskite layer from external moisture and oxygen, which would degrade the device. Therefore, the spiro-OMeTAD HTL should be further optimized to improve the corresponding device stability.

PMMA is a widely used material in PSCs; for example, PMMA is used as an encapsulation material to protect the perovskite layer against oxygen and moisture [17,18,19]. In Li’s report, a thin PMMA layer was added between the electrode and TiO_2_ ETL, and the corresponding PSC acquired apparent stability enhancement. In Snaith’s work, P3HT/SWNTs/PMMA was used to replace spiro-OMeTAD, which exhibited better water and heat stability [20]. However, it should be noted that the device efficiency in their work was not high enough. It is supposed that the electrical conductivity of PMMA is poor, which may negatively affect carrier transport when the complete PMMA layer is inserted into the PSC. Additionally, the PMMA solvent may be harmful to organic HTMs, such as spiro-OMeTAD and PTAA. Inorganic HTMs can combine with PMMA to act as a protective layer. However, inorganic HTMs often need a high annealing temperature, which may not be suitable to form a protective layer on perovskite materials. Therefore, it is important that we pay much attention to the stability of PSCs.

In this work, PMMA is dissolved into the spiro-OMeTAD solvent with different concentrations. It is shown that the stability of the PSCs increases with the PMMA content. When the content of PMMA exceeds the upper limit, the PCE of the device sharply decreases. An optimal content of PMMA exists in the spiro-OMeTAD solvent to keep the balance between long-term stability and high PCE. Finally, we realize a high PCE, over 21%, with improved device stability. The device efficiency can maintain 77% of the original value for PSCs with a PMMA-doped spiro-OMeTAD hole transport layer, under a natural air environment (RH = 40%) for more than 80 days.

## 2. Materials and Methods

Materials: Isopropanol (99.99%), N,N-dimethylformamide (DMF) (99.99%), 4-tert-butylpyridine (99.9%), chloro-benzene (99.9%), and acetonitrile (99.9%) were purchased from Sigma-Aldrich (Shanghai, China) and used as received. Other materials were purchased included MAI (99.9%, Dyesol, Australia), FAI (99.9%, Dyesol, Australia), lead (II) iodide (PbI_2_, 99.999%, Alfa, Haverhill, MA, USA), lead (II) chloride (PbCl_2_, 99.999%, Alfa, USA), and Tin oxide (SnO_2_, 15% in H_2_O colloidal dispersion liquid, Alfa, Haverhill, MA, USA). PMMA (Xi’an Polymer Light Technology Corp, Xi’an, China) and spiro-OMeTAD (Xi’an Polymer Light Technology Corp, Xi’an, China) were also used as received without further purification. The purity of the silver used for the top contact electrode was 99.99%.

SnO_2_ ETL preparation: The ITO substrate was ultrasonically cleaned with detergent solution, acetone, deionized water, and alcohol in sequence. The substrate was first UV−ozone-treated for 15 min. The SnO_2_ solution was diluted with twice the volume of water and spin-coated at 3000 rpm for 30 s. Then the SnO_2_-covered sample was annealed at 150 °C for 30 min in air.

Spiro-OMeTAD solution preparation: 90 mg spiro-OMeTAD powder, 7.65 mg lithium salt, 4.5 mg cobalt salt powder and 10 μL TBP were dissolved in chlorobenzene solvent and then stirred for 12 h. PMMA was added into the spiro-OMeTAD solvent to form the PMMA:spiro-OMeTAD solution.

Solar cell fabrication: PbI_2_ (1.36 m) and PbCl_2_ (0.24 m) were dissolved in DMF solvent and stirred for 2 h at 75 °C. MAI (70 mg) and FAI (30 mg) were dissolved in IPA solvent with 0.9 vol% DMF. Around 75 μL PbX_2_ precursor, preheated to 75 °C, was transferred via pipettes onto the SnO_2_-covered ITO substrate. Then, MAI and FAI mixed solution was spin-coated on top of the dried PbX_2_ layer at 3000 rpm for 45 s. All of the films were thermally annealed on a hot plate at 100 °C for 10 min. Either the spiro-OMeTAD (80 μL) or PMMA:spiro-OMeTAD solution was spin-coated on the surface of the perovskite film at 3000 rpm for 45 s. Lastly, a 100 nm silver layer was deposited through shadow masks via thermal evaporation under a high vacuum. The active area was 0.07 cm^2^.

Device characterization: The morphological measurement of the perovskite layers was taken using scanning electron microscopy (SEM) (JSM-7800F). An X-ray diffraction (XRD) test was conducted on the Bruker D8 Advance XRD. The transmittance and absorption spectra of different samples were recorded with a UV–visible spectrophotometer (Perkin-Elmer Lambda 950). Photovoltaic performances were measured using a Keithley 2400 source meter under simulated sunlight from an XES-70S1 solar simulator, matching the AM 1.5G standard with an intensity of 100 mW/cm^2^. The system was calibrated by an NREL certified silicon reference solar cell. Incident photo-to-current conversion efficiencies (IPCEs) of the PSCs were measured using a solar cell quantum efficiency measurement system (SCS10-X150, Zolix instrument. Co., Ltd., Beijing, China).

## 3. Results

**Figure 1:**
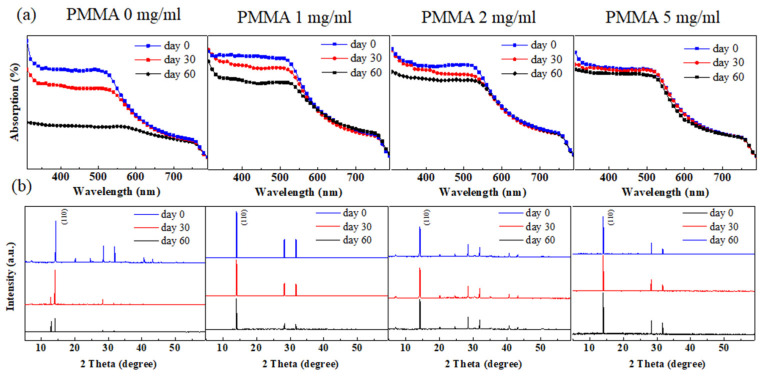
Degradation of perovskite layers with different PMMA (0 mg/mL, 1 mg/mL, 2 mg/mL, and 5 mg/mL): spiro-OMeTAD HTLs. (**a**) Light absorption and (**b**) X-ray diffraction degradation of the perovskite layers over 60 days. The devices were stored in an air environment (RH = 40%), and had a cell structure of ITO/SnO_2_/perovskite/PMMA:spiro-OMeTAD.

It is considered that PMMA-doped spiro-OMeTAD can effectively improve device storage stability, while an overdose of PMMA may adversely decrease the device performance. Thus, there is an optimal concentration of PMMA in the spiro-OMeTAD HTL. In this work, we changed the concentration of PMMA in spiro-OMeTAD from 0 mg/mL to 5 mg/mL. The light absorption degradations of perovskite films with different PMMA:spiro-OMeTAD in the air environment are shown in Figure 1a. It is apparent that the perovskite film protected by the pure spiro-OMeTAD degraded faster than that with the PMMA-doped spiro-OMeTAD. When the storage time was extended to 60 days, the perovskite layer protected by the pure spiro-OMeTAD exhibited a comparatively low light absorption ability. The perovskite films with the PMMA-doped spiro-OMeTAD HTL demonstrated better long-term stability. The greater the concentration of PMMA in spiro-OMeTAD, the weaker the degradation of the perovskite layer. This supports the protective function of PMMA-doped spiro-OMeTAD. A similar phenomenon was also observed in the XRD measurement results in Figure 1b. The perovskite layers all showed intense peaks at 14.2 and 28.5°, indicating the formation of the perovskite crystal structure. For the device with pure spiro-OMeTAD, the perovskite crystal peak exhibited obvious fading, concomitant with the emergence of a well-defined PbI_2_ diffraction peak at 12.6°, which indicates the degradation of the perovskite film. On the contrary, the XRD curves of the perovskite films covered with PMMA:spiro-OMeTAD showed nearly no changes. In addition, no PbI_2_ peaks were detected for all the perovskite XRD curves with devices stored under the same conditions. It can be concluded that the long-term storage stability is enhanced with the increasing content of PMMA in spiro-OMeTAD, as demonstrated in Figure 1.

**Figure 2:**
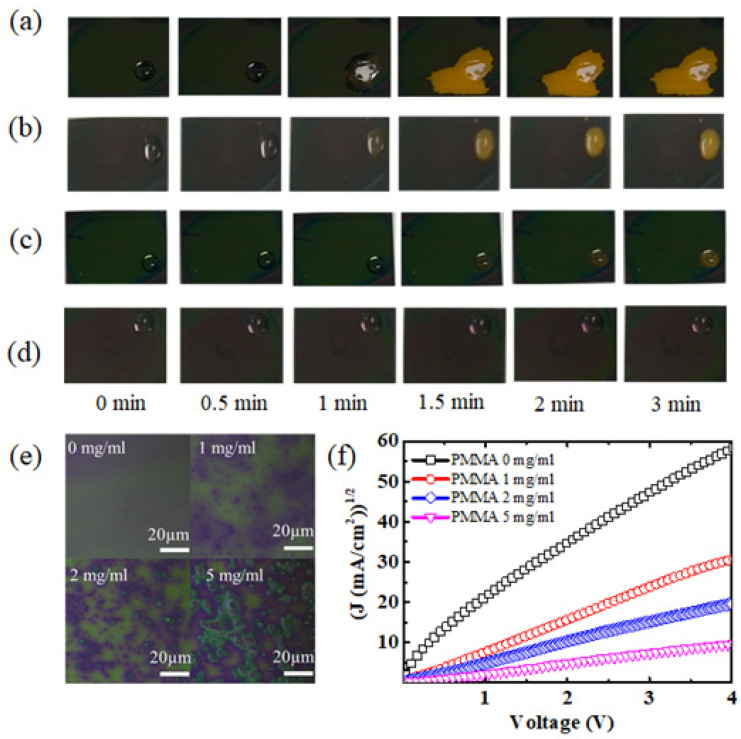
(**a**–**d**) The surface photographs of samples used in the waterproof test with the PMMA (0 mg/mL, 1 mg/mL, 2 mg/mL, and 5 mg/mL)-doped spiro-OMeTAD HTL observed at regular time intervals from 0 min to 3 min. (**e**) The surface topography and (**f**) hole mobility tests of spiro-OMeTAD HTLs doped with different PMMA concentrations (0 mg/mL, 1 mg/mL, 2 mg/mL, and 5 mg/mL).

In previous reports, the spiro-OMeTAD layer was hard to isolate from water due to its inherent amorphous nature and hygroscopic additives, such as Li-salt [21,22]. As presented in Figure 1a,b, with the addition of PMMA, the long-term storage stability of perovskite is improved. To investigate the protective effect of PMMA:spiro-OMeTAD HTL, the waterproof test was developed, as shown in Figure 2a–d, with DI-water being directly dropped onto the perovskite/PMMA:spiro-OMeTAD samples. The sample without PMMA quickly exhibited visible decomposition due to water droplet erosion. In about 1 min, the perovskite layer changed to a yellow color, indicating perovskite film decomposition to PbI_2_. When the doping content of PMMA was increased to 1 mg/mL and 2 mg/mL, the decomposition time of the perovskite layer was delayed by1.5 min and 3 min, respectively, due to the inhibition of water droplet diffusion. In Figure 2d, the perovskite layer almost remained unchanged with time. Therefore, the combined HTL exhibited a better watertight nature with PMMA-doped spiro-OMeTAD. Moreover, The water-resistant properties of PMMA:spiro-OMeTAD-based samples were significantly improved with increased PMMA concentration. The corresponding perovskite layer showed much slower visible degradation and a stronger inhibition of water droplet diffusion. Thus, this result proves that the waterproof characteristics of a PMMA:spiro-OMeTAD combined layer should improve the long-term storage of solar cells.

To further investigate the protective function of PMMA, we carried out a surface topography test of different HTLs. As shown in Figure 2e, the pure spiro-OMeTAD HTL without PMMA is one green, smooth plane. With the addition of PMMA, the surface of spiro-OMeTAD appears uneven and gradually connects into another plane at the content of 5 mg/mL. It is estimated that the unevenness is due to PMMA, which causes the hydrophobic characteristic of spiro-OMeTAD HTL, and further protects perovskite films. It is shown that the surface morphology of the HTLs was greatly changed. An AFM test showed that with the addition of PMMA, the roughness of the PMMA:spiro-OMeTAD surface was improved, which may affect the metal electrode deposition. In addition, PMMA addition may produce a negative influence on the conductivity of HTLs. Both these factors can cause a decrease in the final PCE.

To investigate the effect of PMMA on hole mobility, we carried out measurements using the space-charge-limited-current (SCLC) method, with a structure of ITO/PEDOT:PSS/spiro-OMeTAD/MoO_3_/Au [23,24,25,26,27,28], as shown in Figure 2f. The current density is expressed by the Mott–Gurney Law, as follows:

(1) $$J=\frac{9}{8}\mu \varepsilon \varepsilon_{0}\frac{V^{2}}{L^{3}}$$

where *µ* is the free carrier mobility, *ε* and *ε*_0_ are relative and vacuum dielectric constants, respectively, *V* is the applied voltage, and *L* is the distance between the ITO and Ag electrodes. Both sides of this equation can be square rooted to give the following:

(2) $$\sqrt{J}=\sqrt{\frac{9\mu \varepsilon \varepsilon_{0}}{8L^{3}}}V$$

The change in *J*^1/2^ with *V* is linear. From this, the hole mobility *μ* can be obtained. A hole mobility of 4.66 × 10^−4^ cm^2^ V^−1^ s^−1^ was calculated for the pure spiro-OMeTAD film. Meanwhile, comparable hole mobilities of 1.67 × 10^−4^ cm^2^ V^−1^ s^−1^ and 1.02 × 10^−4^ cm^2^ V^−1^ s^−1^ were achieved for the PMMA-doped spiro-OMeTAD HTLs, with doping concentrations of 1 mg/mL and 2 mg/mL, respectively. When the concentration reached 5 mg/mL, the hole mobility decreased by an order of magnitude to 1.8 × 10^−5^ cm^2^ V^−1^ s^−1^, indicating poor hole transport ability. The hole mobilities calculated from the SCLC model are similar to previously reported values, indicating the accuracy of these results. It can be inferred that the low-dose PMMA (1 and 2 mg/mL) has nearly no negative effect on the hole mobility of spiro-OMeTAD. However, an overdose of PMMA in spiro-OMeTAD will greatly degrade the hole mobility.

**Figure 3:**
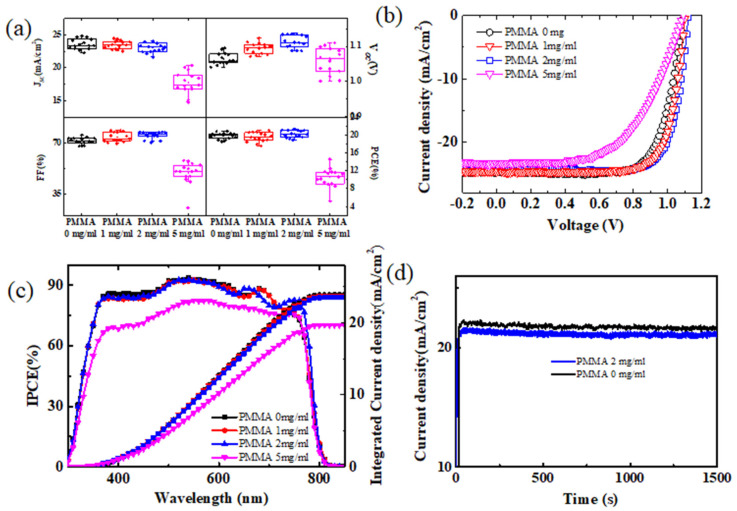
(**a**) Comparison of PSCs’ performance parameters, as follows: short-circuit current density (*J_SC_*), open-circuit voltage (*V_OC_*), fill factor (FF), and power convention efficiency (PCE). All results are based on 15 devices under the same working conditions. (**b**) The *JV* curves of the best devices with different PMMA doping concentrations from 0 mg/mL to 5 mg/mL, and (**c**) incident photon-to-electron conversion efficiency (IPCE) curves of the best devices and corresponding integrated current densities, (**d**) the stabilized current density outputs of solar cells with 0 mg/mL and 2 mg/mL PMMA at the maximum power point (MPP).

As shown in Figure 3a, when the concentration of PMMA is in the range of 0 mg/mL to 2 mg/mL, the *J_SC_* parameters exhibit a slight downward trend. The *V_OC_* and FF show different changing trends, and these values increase with the increasing PMMA concentration. Finally, solar cells reached the highest PCE at 2 mg/mL PMMA concentration. It is shown that moderate PMMA improves the device PCE. When the concentration rises to 5mg/mL, *J_SC_*, FF, and PCE start to decrease rapidly. The decreases in *J_SC_* and FF are mainly caused by the transport carriers being blocked by the high concentration of PMMA. The highest PCE of solar cells based on the pure spiro-OMeTAD HTL was 20.6%, accompanied by *J_SC_* = 24.3 mA/cm^2^, *V_OC_* = 1.11 V, and FF = 77. The best performance of the PSCs was only slightly affected by the low PMMA doping. Moreover, the highest PCE of the devices based on 1 mg/mL (2 mg/mL) PMMA-doped spiro-OMeTAD HTL was 20.9% (21.2%), with the *J_SC_* = 24.0 mA/cm^2^, *V_OC_* = 1.12 V, and FF = 78 (*J_SC_* = 23.9 mA/cm^2^, *V_OC_* = 1.13 V, and FF = 78). When the PMMA concentration reaches 5 mg/mL, the device performance is seriously degraded. Addiotnally, the highest PSC dropped to 9.6%, with the *J_SC_* = 19.5 mA/cm^2^, *V_OC_* = 1.11 V, and FF = 45. Owing to the lack of π-conjugation, PMMA inhibits transport carriers, and usually acts as an insulator for its self-connecting plane. Consequently, the devices with 5 mg/mL PMMA exhibited low performance. The IPCE curves of the best devices and corresponding integrated current densities are displayed in Figure 3c. The devices with 0 mg/mL, 1 mg/mL, and 2 mg/mL PMMA exhibited a very similar absorption in the visible light wavelength (300–800 nm), indicating that PMMA had nearly no effect on light absorption. Moreover, the corresponding integrated current densities were 23.8 mA/cm^2^, 23.6 mA/cm^2^, and 23.5 mA/cm^2^, respectively, which proves the accuracy of the above *JV* curves. The stabilized power output at working state is one important parameter to measure the PSCs’ performance. Figure 3d shows the stabilized current density outputs of solar cells with 0 and 2 mg/mL PMMA at the MPP. The PSC exhibited stabilized high performance, with efficiencies of 21.1% with PMMA addition. The device without PMMA also displayed a steady-state output PCE of 20.4%. It was found that 2 mg/mL PMMA is the optimal concentration.

Based on the above work, PMMA-doped spiro-OMeTAD exhibits a better protective function than pure spiro-OMeTAD on the perovskite layer. To quantify the protective effect, degradation parameters of the best cells with and without PMMA addition were measured, and are exhibited in Table 1. The *JV* curves of these devices were measured and the corresponding PCEs are shown in Figure S1.

As shown in Figure S1 and Table 1, the devices without PMMA addition were stored and tested after storage for 0, 20, 40, and 80 days in the air environment. It should be noted that the stability test followed the ISOS-D1 test as a reference [29]. Compared with the obvious decrease in efficiency from 20.6% to 9.0%, the *V_OC_* of the solar cells exhibited a weaker drop. At the very beginning, the *V_OC_* of the device was 1.11 V, and as the storage time extended to 20, 40, and 80 days, the *V_OC_* fell to 1.10 V, 1.10 V, and 1.08 V, respectively. Another parameter, *J_SC_*, also showed an apparent decrease over time, with values decreasing from the initial 24.3 mA/cm^2^ to 23.7 mA/cm^2^, 20.5 mA/cm^2^, and 15.3 mA/cm^2^ after the storage times of 20, 40, and 80 days. As we have proved with the above results, the perovskite crystal breaks down in the storage process when the PbI_2_ and PbCl_2_ exist in the light absorption layer. The destroyed perovskite crystal vacancy and PbI_2_ (PbCl_2_) can cause the recombination of carriers, accumulation of space charges, and a further decrease in current density, with the introduction of more defects. In addition to the drop in *J_SC_*, the FF also exhibited a distinct decrease from the original 0.76 to 0.71, 0.63, and, finally, 0.55.

The *JV* curves of the devices with PMMA (2 mg/mL): spiro-OMeTAD displayed a relatively negligible change when stored under the same conditions. The *V_OC_* barely changed, ranging from 1.13 to 1.13, 1.12, and, ultimately, 1.12 V, after the storage times of 0, 20, 40, and 80 days. The values for *J_SC_* were estimated to be 23.9, 23.6, 22.7, and 21.4 mA/cm^2^. The FF also exhibited relatively small changes, with values of 0.78, 0.74, 0.70, and 0.67. The PCE of the solar cells with the addition of PMMA fell from 21.2% to 16.3%, maintaining 77% of its original value after being stored in a natural air environment for 80 days. The long-term stability enhancement of the PMMA:spiro-OMeTAD-based devices is mainly attributed to the protective effect of water diffusion into the light absorption layer. The function of PMMA-doped spiro-OMeTAD was also confirmed by the results from a recent study [30].

**Figure 4:**
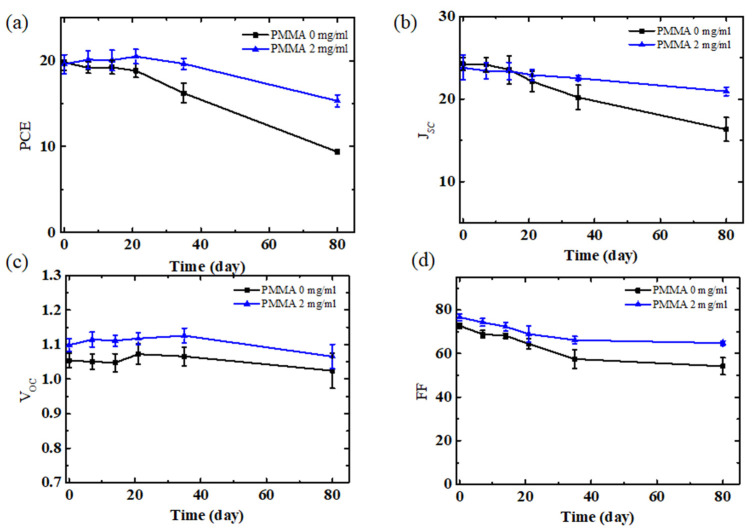
The statistical device stabilities of (**a**) PCE, (**b**) *J_SC_*, (**c**) *V_OC_*, and (**d**) FF of spiro-OMeTAD and PMMA (2 mg/mL): spiro-OMeTAD-based cells. These devices were kept in the air environment with RH = 40% for 80 days.

To further demonstrate the accuracy of our results, the change in the parameter statistics based on 15 devices are presented in Figure 4a–d. The PSCs without PMMA exhibited an average decrease in PCE, maintaining 47% of the initial value after 80 days. On the contrary, the PMMA-treated cells exhibited better efficiency stability, maintaining 78% of the initial value under the same conditions.

**Figure 5:**
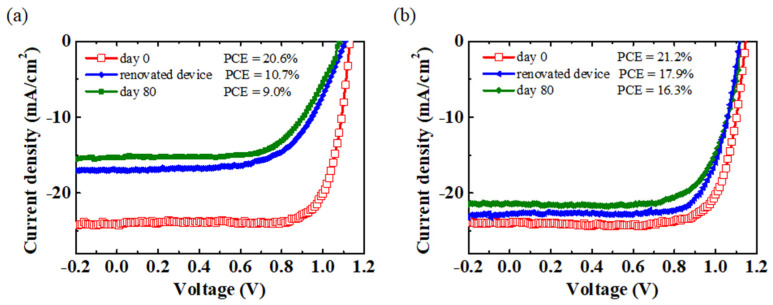
The *JV* curves change in (**a**) spiro-OMeTAD and (**b**) PMMA (2 mg/mL): spiro−OMeTAD based original, long-term storage and renewed devices in the air environment.

To eliminate the effect of spiro-OMeTAD and Ag electrode degradations on device performance under long-term storage conditions, we renovated the devices that had been stored for 80 days by replacing the damaged spiro-OMeTAD with fresh spiro-OMeTAD, and investigated the *JV* characteristics of the renovated devices. The renovating process is shown in Figure S2, with the original Ag electrode removed by the 3M tape and the damaged spiro-OMeTAD removed by CB washing. Then, the fresh spiro-OMeTAD was spin-coated on the perovskite film, followed by the deposition of a new Ag electrode. Finally, the *JV* characteristics of the original, long-term storage and renewed devices are presented in Figure 5. The performance of the renewed devices showed an obvious improvement compared to the long-term storage (80 days) devices, although it is well below the performance of the original devices. These results indicate that the degradation of spiro-OMeTAD itself is not the main factor causing instability. Silver migration and water erosion induce the decomposition of perovskite film, which cannot be recovered by the replacement of spiro-OMeTAD and Ag film. Additionally, the increase in PCE might be due to the restoration of spiro-OMeTAD, and the contact between spiro-OMeTAD and perovskite film. Based on the above results, it can be confirmed that PMMA doping is helpful to improve the long-term storage stability of photovoltaic devices by protecting the perovskite film.

## 4. Conclusions

In conclusion, PSCs show long storage stability improvement with the addition of PMMA in spiro-OMeTAD. The devices based on the PMMA:spiro-OMeTAD HTL exhibited a high PCE of 21.2%, similar to the devices without PMMA, which indicates that PMMA has no negative effect on PCE. The PMMA:spiro-OMeTAD-based devices show excellent long-term stability, maintaining 77% of their original PCEs after being stored for 80 days in the air environment. We provide evidence that PMMA plays a positive role in improving the moisture- and oxygen-resistant ability of PSCs.

## Figures and Tables

**Table 1:** Parameters of PSCs with and without PMMA-doped spiro-OMeTAD in the air environment.

PSC-Day	*J_SC_* (mA/cm^2^)	*V_OC_* (V)	FF (%)	PCE (%)
Without PMMA-0	24.3	1.11	76	20.6
Without PMMA-20	23.7	1.10	71	18.7
Without PMMA-40	20.5	1.10	63	14.2
Without PMMA-80	15.3	1.08	55	9.0
With PMMA-0	23.9	1.13	78	21.2
With PMMA-20	23.6	1.13	74	19.8
With PMMA-40	22.7	1.12	70	17.6
With PMMA-80	21.4	1.12	67	16.3

## Data Availability

The data presented in this study are available on request from the corresponding author.

## Supplementary Materials

The following are available online at https://www.mdpi.com/article/10.3390/polym14020343/s1: Figure S1: *JV* curves of PSCs stored for a long time; Figure S2: The procedures of renovating long-term stored PSCs.
