# Supplementary material for: Stability Improvement of Perovskite Solar Cells by the Moisture-Resistant PMMA:Spiro-OMeTAD Hole Transport Layer
Source: Polymers (Basel). 2022 Jan 17;14(2):343. doi: 10.3390/polym14020343 (PMC8782036; doi:10.3390/polym14020343)
Supplement: Supplementary file 1 [file polymers-14-00343-s001.zip › polymers-1494993-supplementary.pdf]

polymers

MDPI

# Support Information

*Article*

# Stability Improvement of Perovskite Solar Cells by the Moisture-Resistant PMMA:Spiro-OMeTAD Hole Transport Layer

**Shaohua Ma [1,†], Shangzheng Pang [2,†], Hang Dong [2], Xiaoping Xie [3], Gang Liu [3], Peng Dong [3], Dawei Liu [3], Weidong Zhu [2], He Xi [2], Dazheng Chen [2,*], Chunfu Zhang [2,*] and Yue Hao [2]**

[1] Key Laboratory of Micro/Nano Systems for Aerospace, Ministry of Education, Northwestern Polytechnical University, Xi'an 710072, China; mashaohua@spic.com.cn
[2] Wide Bandgap Semiconductor Technology Disciplines State Key Laboratory, Shaanxi Joint Key Laboratory of Graphene, School of Microelectronics, Xidian University, Xi'an 710071, China; szpang@stu.xidian.edu.cn (S.P.); donghangxd@163.com (H.D.); wdzhu@xidian.edu.cn (W.Z.); hxi@xidian.edu.cn (H.X.); yhao@xidian.eud.cn (Y.H.)
[3] Qinghai Huanghe Hydropower Development Co., Ltd., Xining 810008, China; xiexiaoping@spic.com.cn (X.X.); liugang@spic.com.cn (G.L.); dongpeng@spic.com.cn (P.D.); liudawei02@spic.com.cn (D.L.)
* Correspondence: dzchen@xidian.edu.cn (D.C.); cfzhang@xidian.edu.cn (C.Z.)
† These authors contributed equally to this work.

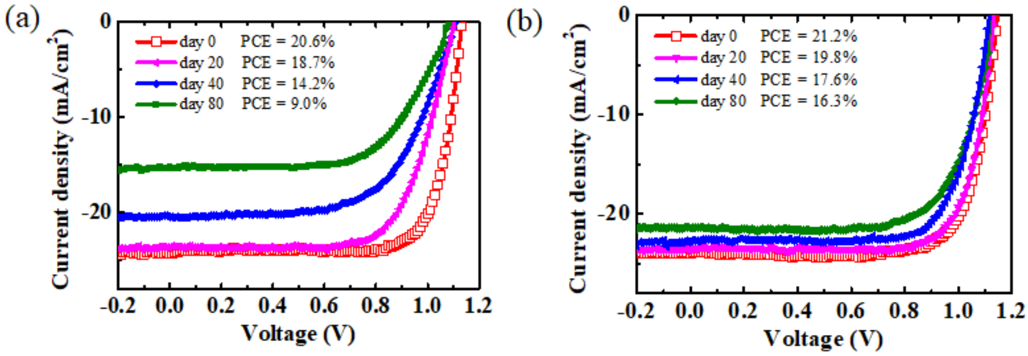


**Figure S1.** *JV* curves of PSCs stored for a long time of (**a**) pure spiro-OMeTAD and (**b**) PMMA (2 mg/mL): spiro-OMeTAD based cells kept in the air atmosphere at RH = 40% for 80 days.

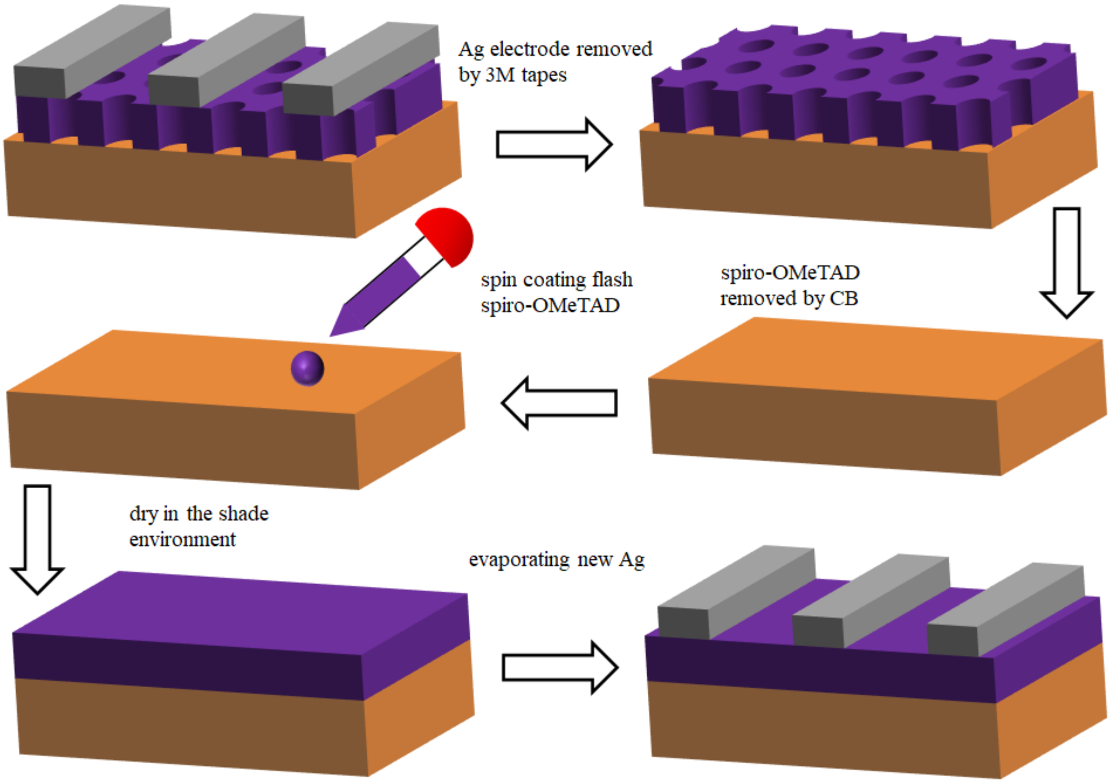


**Figure S2.** The procedures of renovating long-term stored PSCs. The gray layer represents the Ag electrode, the purple layer represents the spiro-OMeTAD and the orange layer represents perovskite and other support layers.
